# Skin transient receptor potential channels expression and microvascular reactivity to cooling in primary and secondary Raynaud's phenomenon

**DOI:** 10.14814/phy2.70557

**Published:** 2025-09-10

**Authors:** A. Guigui, E. Hodaj, J. Rendu, M. Bidart, G. Petre, H. Pluchart, C. Coutton, B. Coste, J. L. Cracowski, S. Blaise, M. Roustit

**Affiliations:** ^1^ Univ. Grenoble Alpes, Inserm, CHU Grenoble Alpes, HP2 Grenoble France; ^2^ Univ. Grenoble Alpes, Inserm, CHU Grenoble Alpes, CIC 1406 Grenoble France; ^3^ Univ. Grenoble Alpes, Inserm, U1216, CHU Grenoble Alpes, Grenoble Institut Neurosciences Grenoble France; ^4^ UM Génétique Moléculaire: Maladies Héréditaires et Oncologie University Hospital Grenoble Alpes Grenoble France; ^5^ INSERM U1209, CNRS UMR 5309, Institute for Advanced Biosciences, Grenoble Alpes University Grenoble France; ^6^ UMR1205, Brain Tech Lab Grenoble Alpes University Grenoble France; ^7^ UM de Génétique Chromosomique University Hospital Grenoble Alpes Grenoble France; ^8^ Pharmacovigilance Unit, CHU Grenoble Alpes Univ. Grenoble Alpes Grenoble France; ^9^ Department of Vascular Medicine, CHU Grenoble Alpes Univ. Grenoble Alpes Grenoble France

**Keywords:** microcirculation, Raynaud's phenomenon, systemic sclerosis, TRP

## Abstract

Temperature‐sensitive Transient Receptor Potential (TRP) channels contribute to modulating skin vascular tone. Their role in Raynaud's Phenomenon (RP) remains unknown. We aimed to investigate TRPs expression in the skin, along with microvascular reactivity to cooling in patients with primary and secondary RP, compared with healthy subjects. Skin blood flow was measured in 10 regions of interest on the dorsum of the hand before, during, and after a 30‐min cooling at 8°C. Skin biopsies were performed from a subset of participants at the distal phalanx before and after cooling. RNAs were extracted, sequenced, and TRPs mRNA expression quantified. TRPs mRNA were successfully quantified, but no significant differences in their expression were observed between groups, or in response to cooling. There was a decreased perfusion at the distal phalanx over time, in secondary RP compared with primary RP (adjusted *p* = 0.03) or healthy subjects (adjusted *p* = 0.042). Gene expression profiles were more similar between primary RP and healthy subjects than with secondary RP. This study shows an impairment of microvascular reactivity to cold in secondary, but not in primary RP. Transcriptomic data suggest involvement of the NO pathway in secondary RP, while TRP channel expression appears unchanged; however, their function could be impaired, which should be further investigated.

## 1 INTRODUCTION

Raynaud's Phenomenon (RP) is a clinical syndrome corresponding to transient ischemia of the digits triggered by cold, moisture, or emotional stress (Herrick, 2019). It is either primary and benign or secondary to diseases like systemic sclerosis (SSc), a connective tissue disease characterized by vascular alterations, immune system activation, and tissue fibrosis (Trojanowska, 2010). Secondary RP is associated with significant morbidity, including a risk of digital ulceration. To date, most treatments have limited effectiveness, (Khouri et al., 2019) highlighting the need for new therapeutic targets, which requires a better understanding of its pathophysiology.

Transient receptor potential (TRP) channels are a family of cationic channels composed of six transmembrane domains (Latorre et al., 2009). These transmembrane protein complexes play a crucial role in the nervous system. TRPs are also present in vascular walls, where they modulate vascular tone in addition to functioning as molecular thermal sensors. Several TRPs expressed in the skin are sensitive to temperature changes (TRPV1, TRPV3, TRPV4, TRPM8, TRPA1), and contribute to vascular reactivity and remodeling (Earley & Brayden, 2015; Johnson et al., 2009; Martín‐Bórnez et al., 2020; Zholos, 2010).

Cutaneous microvascular dysfunction is a hallmark of RP pathophysiology. Indeed, abnormal microvascular reactivity to cooling has been observed both in primary RP (Flavahan, 2015; Roustit et al., 2011) and in secondary RP (Hughes et al., 2020). The mechanisms underlying this dysfunction remain partly unknown. Whether temperature‐sensitive TRP channels are involved in abnormal reactivity to cooling in RP has never been explored in primary RP, while preliminary data suggest increased expression of functional TRPM8 in the skin of patients with SSc (American College of Rheumatology Empowering Rheumatology Professionals, 2013) However, whether this expression is associated with abnormal microvascular reactivity to cold has not yet been investigated.

Our objective was therefore to explore the expression of thermosensitive TRPs in the skin, along with microvascular reactivity to a cooling test, in primary and secondary RP, compared with healthy subjects.

## 2 METHODS

### 2.1 Study design and population

Study participants were recruited from two prospective, pathophysiological studies comparing microvascular function in response to cooling among healthy subjects, patients with primary RP, and patients with RP secondary to SSc (NCT01743612). A subgroup of participants was proposed to participate in the second study, which included skin biopsies for transcriptional analyses (NCT03211325).

All participants were over 18 years old. Primary or secondary RP was diagnosed according to the criteria of LeRoy and Medsger (LeRoy & Medsger, 2001). Color charts were used to confirm diagnosis and detail the topography of RP. Patients with SSc were recruited through the vascular medicine department of Grenoble Alpes University Hospital and met the EULAR/ACR classification criteria (van den Hoogen et al., 2013). Primary RP patients and healthy participants were recruited from the general population via newspaper advertisements and were matched to SSc patients by sex and age (+/− 5 years). Subjects who were smokers, pregnant, or had a severe concomitant pathology noted on examination as active cancer, liver failure, or a history of myocardial infarction within the last 5 years or angina, or who were in an exclusion period for other studies were not eligible. Subjects with active ulcers or a history of ulcers were ineligible for skin biopsies.

The Institutional Review Board of Sud‐Est V, Grenoble, France (No. 6705), approved the protocols. Written informed consent was obtained from all subjects before participation.

### 2.2 Cooling test and skin blood flow measurements

**FIGURE 1:**
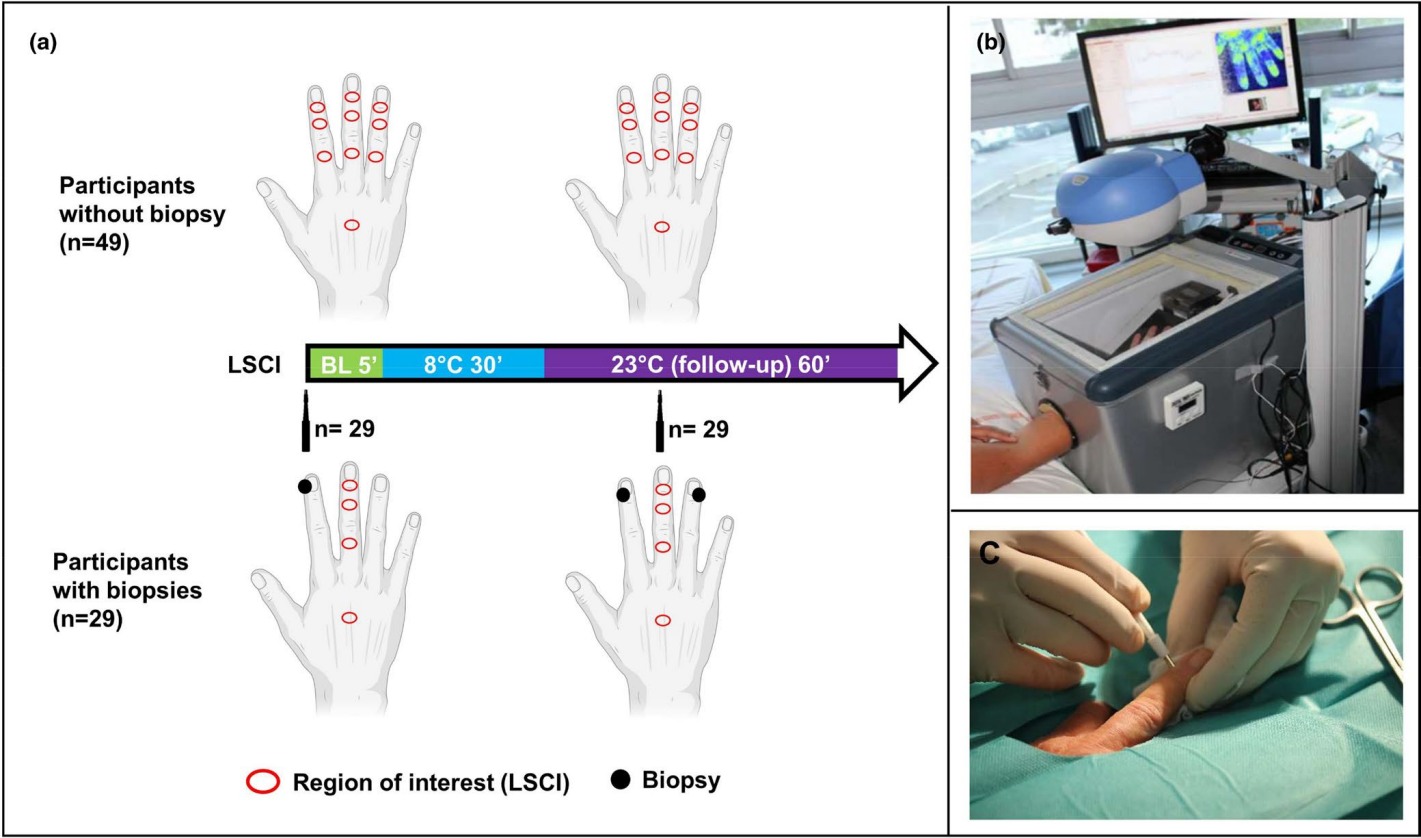
Presentation of the study process. (a) The red circles represent regions of interest, that is, sites where skin blood flow was recorded using LSCI. Black dots represent the sites of skin biopsies for the subgroup of participants in whom biopsies were performed. (b) Photograph of the cooling room and laser speckle contrast imaging. (c) Photograph of a biopsy.

Measurements were performed in a temperature‐controlled room (23+/−1°C). The participant's arm was immobilized with a vacuum cushion to minimize movement artifacts. After a 30‐min acclimation, baseline skin blood flow of the dorsal face of the hand was recorded for 5 min using Laser Speckle Contrast Imaging (Pericam PSI, Perimed, Jarfalla, Sweden). This technique has good spatial and temporal resolution and very good reproducibility. (Cracowski & Roustit, 2020; Roustit et al., 2010) The hand was then inserted into a cooling box (prototype; Perimed, Järfälla, Sweden) at 8 +/−1°C for 30 min. Skin blood flow was recorded for 40–60 min post‐cooling, as previously described (Roustit et al., 2018) (Figure 1).

Throughout the procedure, blood pressure was measured every 5 min, on the opposite arm. Skin blood flow was expressed as cutaneous vascular conductance (CVC), calculated as the ratio between arbitrary perfusion units and mean arterial pressure (AU/mmHg) (Cracowski & Roustit, 2020).

Skin blood flow was analyzed at 10 regions of interest (ROI): one on the dorsum of the hand, and three on the index, middle, and ring fingers (distal, middle, and proximal phalanx). Subjects who had biopsies only had one finger analyzed and therefore 4 ROI in total (Figure 1).

Data were digitized, stored on a computer and analyzed off‐line with signal processing software (Pimsoft 1.1.1; Perimed; Jarfalla, Sweden). Flow was averaged over 3‐min periods, at multiple times of interest (TOIs): baseline, beginning of the cooling test, at 10, 20, and 30 minutes of the cooling test, immediately after cooling, and 10, 20, 30, 40, and 60 min post‐cooling. Data were expressed as percentage change from baseline CVC. In subjects without biopsy, CVC was averaged over 1‐min periods, and areas under the curve (AUC) were computed for both the cooling (30 min), and rewarming (60 min) periods. Details on ROIs and TOIs per subgroup are available in the appendix (Tables S2 and S3).

### 2.3 Skin biopsies

Three skin biopsies were collected from a subgroup of participants in each group: two from fingers and one from the gluteal area as a control site not affected by RP. The two fingers biopsied were randomized between the middle, the ring, and the index of the non‐dominant hand. On the right hand, biopsies were taken from the distal phalanx before cooling (finger 1) and within 10 min post‐cooling (finger 2). Local anesthesia (subcutaneous lidocaine) was applied, and a 3‐mm biopsy punch was used to perform biopsies. Sutures were then realized with non‐resorbable thread. Biopsies were frozen in liquid nitrogen and stored at −80°C before analysis. Stitches were removed 1 week later.

### 2.4 RNA extraction

Skin biopsies were ground mechanically. Then, the long RNA of each biopsy was extracted with the miRVanaTM kit (Ambion, Life technologies, Cat. No. AM1561). Briefly, the extraction was carried out with phenol chloroform which, after stirring and centrifugation, yielded three phases: the RNAs, the proteins, and the genomic deoxyribonucleic acid (DNA).

To purify long RNA on column, 0.3 Volume of Ethanol 100% was added to 1 Volume of Aqueous phase (Ethanol at 25% final). Only long RNAs were retained. These were then eluted with aqueous buffer. The RNA samples were stored at −80°C.

### 2.5 TRP mRNA expression

Then, RNA was quantified with the NanoDrop™ 2000/2000c spectrophotometer (Thermo Fischer Scientific, RRID:SCR_018042) with the absorbance at 260 nm. RNA quality was assessed with an Agilent Eukaryote Total RNA Nano assay in a 2100 Bioanalyzer (Agilent Technologies, Santa Clara, CA, USA). RNAs were then reverse transcribed with the iScriptTM reverse transcription supermix for RT‐qPCR kit (Bio‐Rad, France, Cat No 1708841). Reverse Transciption reactions were adjusted to a final volume of 100 μL with 50 mM Tris buffer (pH 8). Then, qPCR analyses were performed on a CFX96 TouchTM Real‐Time PCR Detection System (Bio‐Rad, France, RRID:SCR_018064), using the SsoAdvancedTM Universal SYBR® Green Supermix kit (Bio‐Rad, France, Cat. No. 172‐5271). The PCR protocol used was: 3 min at 95°C and 40 cycles with 20 s at 95°C and 20 s at 60°C.

PCR primers were designed using the Universal Probe Library Assay Design Center. The efficiency of primers was checked with a standard curve realized with dilution series of cDNA from various tissues S1. We used HPL27 as a housekeeping gene. The primer sequences that we used are described in Supporting Information; Table.

### 2.6 Skin RNA Sequencing

RNAseq was performed on total RNA extracted from the biopsies. rRNA removal was realized with positive polyA+ selection. RNAseq was realyzed with paired end sequencing 2*150 on an Illumina NovaSeq (30 Millions reads per sample, RRID:SCR_016387). FASTQ files were then treated with NFCORE rnaseq bioinformatical pipeline for alignement and differential expression (Ewels et al., 2020). Transcripts with Log2FC <−0.5 or Log2FC >0.5 with a *p* value <0.05 were analyzed. Then, using Cytoscape (Shannon et al., 2003) software v3.9.1 (RRID:SCR_003032) with String app v11.5 (RRID:SCR_005223) (Doncheva et al., 2019) enriched pathway were identified. Only Reactomes pathway, Kegg Pathway, and String Cluster were investigated.

### 2.7 Statistical analysis

Considering the exploratory nature of this study, we estimated the effect size that could be detected with a given sample size before starting the study. We calculated that including 10 subjects per group would provide 80% power to detect an effect size >0.35 (estimated as the variance of the means divided by the within‐group variance), using a linear model with an alpha level of 0.05. Continuous variables were expressed as means and standard deviations, and categorical variables as numbers and percentages. *p*‐values <0.05 were considered significant. One‐way ANOVA (or Kruskal–Wallis test if not applicable) was performed to compare continuous variables between the three groups. Paired comparisons were analyzed with the *t*‐test or Mann–Whitney *U* test when needed.

Mixed effects linear models were used to analyze microvascular reactivity. We included the effect of time, group, skin site, and their interactions; we also included the subject as a random factor. Baseline skin blood flow was used as a covariate. Normality of the distributions was assessed both visually and with the Shapiro–Wilk test. When the data were not normally distributed, Ln transformation was considered. Paired comparisons between groups were performed as post hoc analyses, using Bonferroni correction to take into account the multiplicity of comparisons. Statistical analyses were performed using Jamovi® version 1.6.16.0.

## 3 RESULTS

### 3.1 Skin blood flow measurements

A total of 78 participants were enrolled: 27 healthy subjects, 27 patients with primary RP, and 24 with secondary RP. Among them, biopsies were obtained from 10 healthy subjects, 10 patients with primary RP, and 9 patients with secondary RP (Figure S1). Demographic and clinical characteristics are reported in Table 1.

**TABLE 1:** Population's characteristics.

Characteristics	Groups		
	Healthy subects, *n* = 27	Primary RP, *n* = 27	Secondary RP, *n* = 24
Age (years)	54.1 (9.7)	55.7 (12.5)	60.8 (11.3)
Female, (*n*)	19 (70.4%)	23 (85.2%)	21 (87.5%)
Body mass index (kg)	24.8 (4.3)	23.3 (3.3)	24.9 (3.4)
Mean arterial blood pressure (mmHg)	91.5 (12.5)	89.8 (11.4)	88.6 (12.2)
RP	0 (0)	27 (100%)	24 (100%)
Duration (years)	NA	25.9 (15.1)	13.3 (12.1)
No fingers involved	NA	8.6 (1.9)	8.3 (2.1)
Thumb involved	NA	13 (48%)	12 (50%)
Other locations (feet, nose, ears)	NA	9 (33.3%)	5 (20.8%)
RP frequency‐winter, *n* (%)			
>5/day	NA	1 (3.7%)	8 (33.3%)
1–5/day	NA	16 (59.3%)	8 (33.3%)
1/week to 1/day	NA	8 (29.6%)	7 (29.2%)
<1/week	NA	2 (7.4%)	1 (4.2%)
RP frequency‐summer, *n* (%)			
>5/day	NA	0 (0%)	2 (8.7%)
1–5/day	NA	3 (11.1%)	6 (26.1%)
1/week to 1/day	NA	9 (33.3%)	9 (39.1%)
<1/week	NA	15 (55.6%)	6 (26.1%)
Rodnan‐modified score	NA	NA	7.55 (5.7)
Raynaud's conditions score	NA	5.7 (2.1)	4.1 (2.7)
Capillaroscopy pattern: *n* (%) non active/early/active/late	NA	NA	3 (12.5%)/9 (37.5%)/6 (25%)/6 (25%)
Treatment, *n* (%)			
Iloprost	0 (0%)	0 (0%)	1 (4.2%)
Bosentan	0 (0%)	0 (0%)	1 (4.2%)
Calcium blockers	2 (7.4%)	2 (7.4%)	13 (54.2%)
Corticosteroids	0 (0%)	0 (0%)	4 (16.7%)
Other	6 (22.2%)	7 (25.9%)	16 (66.7%)

*Note*: Demographic and clinical characteristics of healthy subjects, Patients with primary Raynaud's phenomenon and systemic sclerosis. Quantitative data are expressed as mean (standard deviation); Qualitative data are expressed as number (percentage).

Abbreviation: NA: not applicable.

Mean CVC for all groups and regions of interest is presented in Table S2. There was no significant difference in baseline skin blood flow between groups at the dorsum of the hand (*p* = 0.483), proximal (*p* = 0.06), middle (*p* = 0.073), or distal phalanxes (*p* = 0.191).

**FIGURE 2:**
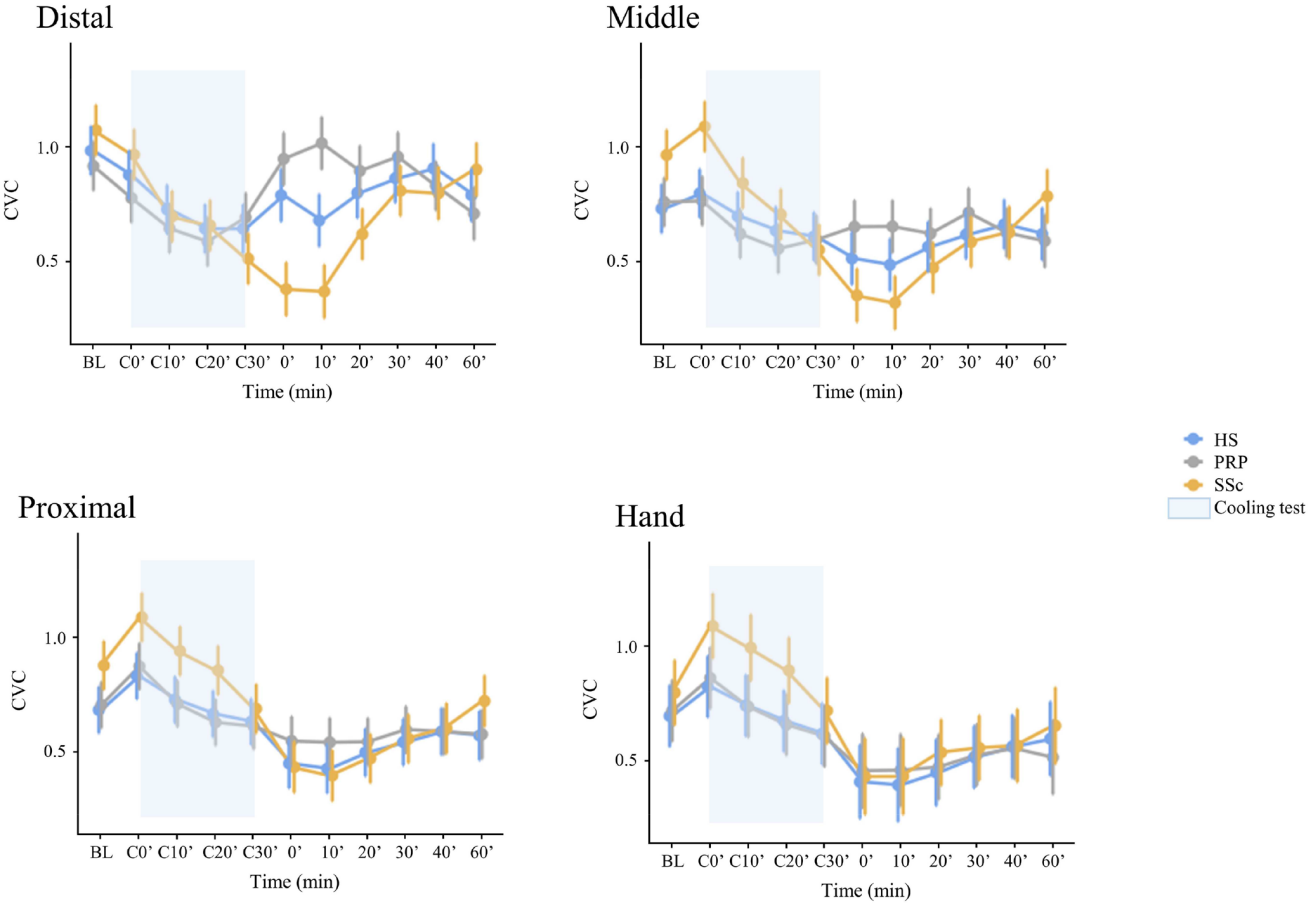
Mean change over time in CVC according to the skin sites and the group of participants. The line graph represents the mean values with standard error bars. CVC is expressed as AU/mmHg.

Overall, significant differences in skin perfusion over time were observed between the different skin sites (i.e., significant time‐group interaction, *p* < 0.001) (Figure 2), as well as a significant site‐group interaction (*p* < 0.001). Post hoc analyses showed that the difference between groups is significant on the distal phalanx, with a lower CVC in patients with secondary RP (0.81 ± 0.57 AU/mmHg), in comparison with those with primary RP (0.87 ± 0.53 AU/mmHg; *p* = 0.03, adjusted for multiple comparisons) or healthy subjects (0.87 ± 0.44 AU/mmHg; adjusted *p* = 0.042). There was no significant difference on the other skin sites. Similarly, there was an effect of time with a significant time‐site interaction (*p* < 0.001) (Figure 2). Details for each time point are provided in Table S2.

When looking at cooling and rewarming periods separately, we observed a significant site‐group interaction (*p* < 0.001) for the AUC (Table S3). This reflected an inversion of the skin perfusion gradient in secondary RP patients compared with the other groups, that is, a higher skin perfusion at the dorsum of the hand in secondary RP patients, but a lower perfusion at distal phalanxes (Figure S2).

Similarly, there was a significant site‐group interaction (*p* < 0.001) for rewarming, with a lower perfusion distally in secondary RP (43.0 AU/mmHg ± 26.7) than in primary RP (55.2 ± 23.3 AU.min/mmHg; adjusted *p* = 0.009).

### 3.2 TRP mRNA expression

All temperature‐sensitive mRNA coding for TRPs sensitive to temperature changes were successfully quantified in the skin. Due to technical issues, biopsies from one healthy volunteer could not be analyzed. No significant difference in their expression was observed between groups at baseline, nor after cooling (Table 2).

**TABLE 2:** TRP mRNA expression.

		Healthy subject	Patients with primary RP	Patients with SSc	*p*‐value
		Median (IQR)	Median (IQR)	Median (IQR)	
TRPA1	Finger–before cooling	0.18 (0.02–0.56)	0.15 (0.06–0.27)	0.33 (0.13–0.86)	0.4381
	Finger–after cooling	0.22 (0.05–0.70)	0.24 (0.11–0.52)	0.28 (0.12–0.90)	0.5225
	*p*‐value	0.8109			
	Control–gluteal area	0.30 (0.01–1.78)	1.27 (0.84–2.35)	1.66 (0.74–3.85)	0.1263
TRPV3	Finger–before cooling	0.45 (0.38–0.59)	0.61 (0.27–0.82)	0.46 (0.23–0.87)	0.7808
	Finger–after cooling	0.46 (0.24–0.57)	0.663 (0.445–0.895)	0.656 (0.219–1.117)	0.277
	*p*‐value	0.2682			
	Control–gluteal area	1.55 (0.82–1.6)	1.10 (0.72–1.25)	2.02 (0.68–3. 20)	0.7124
TRPM8	Finger–before cooling	0.20 (0.06–0.33)	0.29 (0.16–0.41)	0.23 (0.10–0.33)	0.6494
	Finger–after cooling	0.31 (0.06–0.46)	0.38 (0.22–0.53)	0.26 (0.12–0.72)	0.8388
	*p*‐value	0.5074			
	Control–gluteal area	1.25 (0.76–1.42)	1.15 (0.75–1.36)	1.03 (0.74–2.45)	0.9415
TRPV6	Finger–before cooling	0.32 (0.13–0.51)	0.44 (0.32–0.60)	0.34 (0.14–0.89)	0.7201
	Finger–after cooling	0.49 (0.16–0.55)	0.59 (0.38–0.69)	0.42 (0.18–1.20)	0.7727
	*p*‐value	0.355			
	Control–gluteal area	1.35 (0.87–1.49)	1.07 (0.83–1.45)	1.54 (0.69–2.48)	0.8128
TRPV1	Finger–before cooling	0.15 (0.08–0.45)	0.36 (0.18–0.48)	0.38 (0.15–1.02)	0.3487
	Finger–after cooling	0.41 (0.14–0.49)	0.49 (0.20–0.78)	0.37 (0.21–0.94)	0.7785
	*p*‐value	0.5266			
	Control–gluteal area	0.85 (0.15–1.35)	1.03 (0.95–1.30)	1.35 (0.52–2.40)	0.3438
TRPV4	Finger–before cooling	0.15 (0.14–0.55)	0.42 (0.14–0.44)	0.40 (0.18–1.16)	0.419
	Finger–after cooling	0.29 (0.07–0.52)	0.33 (0.09–0.63)	0.58 (0.20–1.37)	0.3002
	*p*‐value	0.5911			
	Control–gluteal area	0.24 (0.10–1.52)	1.02 (0.62–1.29)	1.91 (0.71–3.61)	0.1925

*Note*: *p*‐value from Table 2 are, in row, *p* value between groups and in column, *p*‐value of interaction between groups‐site of biopsies for repeated value (before and after cooling). Data are expressed as median and interquartile range (IQR).

### 3.3 RNA sequencing

Differential gene expression between the three conditions is illustrated in a Venn diagram (Figure S3). The full data set is provided as supplementary data (Data S1). We observed a higher degree of similarity in gene expression between primary RP patients and healthy subjects, in comparison with secondary RP patients. Indeed, 636 genes were underexpressed in biopsies from patients with secondary RP compared to healthy subjects, and 733 compared to patients with primary RP (among which 315 genes are commonly underexpressed in secondary RP patients compared to the two other groups). On the other hand, only 66 genes were underexpressed in biopsies from patients with primary RP compared to healthy subjects.

Similarly, 243 genes were overexpressed in biopsies from patients with secondary RP compared to healthy subjects, and 186 compared to primary RP (76 in common). Finally, 52 genes were significantly overexpressed in biopsies from primary RP patients compared to healthy subjects.

Pathway analyses suggest that two pathways are downregulated in secondary RP, both against primary RP patients and healthy subjects (Table S4): the “nitric oxyde stimulates guanylate cyclase” pathway (HSA‐392154) (Figure S4), and the “extracellular matrix organization” pathway (HSA‐1474244) (Figure S5).

Pathway analysis before vs. after cooling revealed several dysregulations in secondary RP, but none seemed related to microvascular function or matrix reorganization (Data S2).

## 4 DISCUSSION

The present study shows impaired digital vascular reactivity to local cooling in secondary RP (SSc) patients, compared to healthy subjects and patients with primary RP. However, contrary to our initial hypothesis, these differences do not appear to be related to differences in TRP mRNA expression. On the other hand, we observed impaired gene expression of the NO pathway in secondary, but not in primary RP, which is consistent with the functional results.

The skin, as a key thermoregulatory organ, has a microvasculature that is particularly reactive to temperature changes. More specifically, local cooling leads to an initial vasoconstriction, followed by a transient vasodilation and finally a prolonged vasoconstriction (Johnson & Kellogg, 2010). The mechanisms underlying local cooling‐related vasoconstriction involve inhibition of the NO pathway, through reduced NO and NOS production, as well as afferent sensory nerves and increased translocation of postsynaptic α2c‐adrenoreceptors via the RhoA‐RhoKinase pathway (Flavahan, 2015). On the other hand, the dilation induced by local cooling, which attenuates tissue damage from excessive vasoconstriction, involves non‐NO and non‐PGI2‐dependent mechanisms (Flavahan & Flavahan, 2020). Recent experimental studies suggest increased direct communication between endothelium and smooth muscle cells and the involvement of the EDHF‐dependent pathway via SK3 potassium channels (Chang et al., 2023; Flavahan & Flavahan, 2020).

Several studies have explored microvascular dysfunction in response to cooling in patients with RP to better understand the pathophysiology of this syndrome. In primary RP, we have previously described abnormal cutaneous microvascular reactivity to local cooling on the dorsum of the finger, partly depending on sensory nerves (Roustit et al., 2011) Experimental work has suggested an involvement of α2‐adrenoreceptors. (Flavahan, 2015; Freedman et al., 1995). In the present study, however, we did not observe any difference in microvascular reactivity between patients with primary RP and healthy participants. This is probably explained by the different cold challenge that we used. Indeed, here we exposed the whole hand to cooled air in a temperature‐controlled chamber. Although this test is not considered a valid surrogate for RP severity (Guigui et al., 2023), such regional cooling seems a better pathophysiological model, closer to the conditions in which RP typically occurs. In addition, it permits assessment of skin blood flow during and after cooling (rewarming period) (Roustit et al., 2018). However, it probably involves different mechanisms than those triggered by direct contact of a cooled laser‐doppler probe, which we had used previously. The absence of abnormal skin microvascular reactivity in primary RP is consistent with the findings of previous studies that assessed rewarming after a water cold challenge, measuring perfusion with LSCI or Laser Doppler (Della Rossa et al., 2013; Grattagliano et al., 2010). There was abnormal reactivity in secondary RP versus primary RP and healthy subjects, mostly driven by abnormal rewarming in patients with SSc. This is also consistent with previous work (Herrick et al., 2020; Rosato et al., 2011). It is worth noting that this difference only reaches significance on the distal phalanx. At other skin sites, there was a trend toward higher baseline flux in SSc, which is also consistent with previous results, (Della Rossa et al., 2013) and potentially attributable to vasodilator use (especially calcium channel blockers).

These results on microvascular reactivity are consistent with the downregulated NO pathway revealed by RNA sequencing from digital skin biopsies and prior literature. Asymmetric dimethylarginine (ADMA), an inhibitor of endothelial NO synthase, is increased in patients with secondary RP compared to primary RP or healthy subjects (Blaise et al., 2009; Dooley et al., 2006; Rajagopalan et al., 2003). The production of NO is altered in SSc. eNOS is decreased while iNOS is increased, making the NO produced more harmful than protective. Indeed, the NO produced by iNOS is deleterious because it is transformed into peroxynitrite (Matucci Cerinic & Kahaleh, 2002), a reactive oxidizing species (ROS). This compound is considered to be involved in the development of the disease by stimulating pro‐inflammatory and pro‐fibrotic cytokines. (Blaise et al., 2009; Gabrielli et al., 2012; Vona et al., 2018) ROS levels were found higher in fibrotic skin than in non‐fibrotic skin of SSc patients and significantly higher than in skin from healthy subjects (Bourji et al., 2015). Abnormal metabolism of NO could reflect the endothelial dysfunction and vasculopathy of SSc patients. Indeed, the NO pathway was significantly downregulated in patients with SSc‐related RP compared to primary RP or healthy subjects.

Altogether, our findings confirm previous experimental data showing that the pathophysiology of RP differs between primary and SSc‐related RP. However, the temperature sensor which may trigger RP in humans has not yet been identified. As channel receptors sensitive to temperature changes involved in microvascular function (Aubdool et al., 2014; Bautista et al., 2007; Zhang et al., 2023) TRP channels were good candidates: TRPM8 and TRPA1 as cold sensors and TRPV1, TRPV3, and TRPV4 as heat sensors. Yet, our results do not support these hypotheses. Specifically, TRPM8 channels, which are activated by menthol or cold, are present on human skin and increase skin blood flow via activation of muscarinic receptors or the NO pathway (Johnson et al., 2009). They also demonstrate a thermosensor role on rats and increase core temperature. However, studies on rodents suggest that TRPM8 activation in response to cold regulates core temperature through thermogenesis, and not via an impact on skin blood flow (Almeida et al., 2012; Knowlton et al., 2011; Tajino et al., 2011). Besides TRPM8, we found no between‐group difference in the expression of any TRP explored, and no effect of cooling on their expression.

Our study had several limitations; first, the number of subjects per group varied across measurement times, and although the groups remained balanced throughout the analysis, we may have lacked power to detect small changes. The substantial between‐subject variability may also explain a part of the lack of significant differences, as reflected by the large interquartile ranges. Second, we did not consider the temperature of the room in our statistical model, due to an already important number of parameters. In a study in 6 centres in the United Kingdom, Wilkinson et al. confirmed that a small variation of the temperature is acceptable for LSCI and thermography results (Wilkinson et al., 2018). Although subjects had no instruction regarding clothing, experiments were conducted in a temperature and humidity‐controlled room for at least 30 min before the evaluation of the microcirculation by LSCI. We therefore expect a limited impact on our conclusions. Third, our RNA‐centric analysis technique allowed us to objectify local transcriptional changes only. Yet, the potential TRP channel involvement in the pathophysiology of Raynaud's phenomenon cannot be ruled out on the basis of mRNA expression data alone. Since these channels are fairly stable, we may expect post‐translational alterations or changes in plasma membrane trafficking. TRP channel function could notably be regulated by mechanisms independent of transduction or translation. We might also suppose a slower phenomenon of transcription modulation in response to cold and that our 30‐min cooling was too short to trigger such modifications. But in our experience, longer exposures to cold are less well accepted by participants.

Similarly, although our data are consistent with previous work suggesting impaired NO pathway in secondary RP, this study does not fully capture the role of NOS and NO in the pathophysiology of RP. In particular, eNOS activity is highly dependent on protein phosphorylation, protein–protein interactions, co‐factors availability, and oxidative stress, all of which are not captured in bulk RNAseq analyses. In addition, there is cross‐talk between temperature‐sensitive TRP channels and the NO pathway through calcium‐dependent signaling, which our approach does not permit to detect.

Finally, our findings only pertain to the TRP channels examined. We cannot exclude involvement of other TRP family members that were not investigated in this study.

In conclusion, this study shows an impairment of microvascular reactivity to cold in secondary, but not in primary RP. Our data suggest dysregulated NO pathway in secondary RP. Although transcriptomic analyses did not reveal significant changes in TRP channel mRNA expression, this does not exclude their potential involvement in RP, as their function is regulated at multiple post‐transcriptional levels. Further functional and proteomic investigations will be necessary to clarify the molecular mechanisms underlying Raynaud's phenomenon.

## AUTHOR CONTRIBUTIONS

A. Guigui, E. Hodaj, B. Coste, G. Petre, and H. Pluchart contributed to acquisitions. A. Guigui and M. Roustit contributed to the interpretation of data, analysis and drafting the work. J. Rendu, M. Bidart, and C. Coutton contributed to the interpretation of data. M. Roustit, S. Blaise, and J. L. Cracowski contributed to the conception and design. All authors revised the manuscript.

## FUNDING INFORMATION

This study was funded by the Association des Sclérodermiques de France and the Grenoble Alpes University Hospital.

## CONFLICT OF INTEREST STATEMENT

Authors have no conflict of interest to declare for this study.

## ETHICS STATEMENT

The Institutional Review Board of Sud‐Est V, Grenoble, France (No. 6705), approved the protocols. Written informed consent was obtained from all subjects before participation.

## Supporting information


Data S1.

## Data Availability

Data are available on request to the corresponding author. Supporting Information and RNA sequencing data are available on supplementary data: https://osf.io/8yf5x/?view_only=59b4d5a5d0ba4400956e41005c2718bc
