# Supplementary material for: Skin transient receptor potential channels expression and microvascular reactivity to cooling in primary and secondary Raynaud's phenomenon
Source: Physiol Rep. 2025 Sep 10;13(17):e70557. doi: 10.14814/phy2.70557 (PMC12422803; doi:10.14814/phy2.70557)
Supplement: Supplementary file 1 — Data S1. [file PHY2-13-e70557-s001.docx]

Skin Transient Receptor Potential Channels expression and microvascular reactivity to cooling in primary and secondary Raynaud’s phenomenon.

Guigui A^1,2^, Hodaj E^2^, Rendu J^3^, Bidart M^4,8^, Petre G^9^, Pluchart H^2^, Coutton C^5,8^, Coste B^2^, Cracowski JL^1,6^, Blaise S^1,7^, Roustit M^1,2^

^1^Univ. Grenoble Alpes, Inserm, CHU Grenoble Alpes, HP2, 38000 Grenoble, France.

^2^Univ. Grenoble Alpes, Inserm, CHU Grenoble Alpes, CIC 1406, 38000 Grenoble, France.

^3^Univ. Grenoble Alpes, Inserm, U1216, CHU Grenoble Alpes, Grenoble Institut Neurosciences, 38000 Grenoble, France.

^4^ UM Génétique Moléculaire: Maladies Héréditaires et Oncologie, University Hospital Grenoble Alpes, 38000 Grenoble, France.

^5^UM de Génétique Chromosomique, University Hospital Grenoble Alpes, 38000 Grenoble, France.

^6^Univ. Grenoble Alpes, Pharmacovigilance unit, CHU Grenoble Alpes, 38000 Grenoble, France.

^7^Univ. Grenoble Alpes, Department of Vascular Medicine, CHU Grenoble Alpes, 38000 Grenoble, France.

^8^INSERM U1209, CNRS UMR 5309, Institute for Advanced Biosciences, Grenoble Alpes University, 38000 Grenoble, France.

^9^UMR1205, Brain Tech Lab, Grenoble Alpes University, Grenoble, 38000, France

Supplementary material

Table of content

[Supplemental figures 3](#_Toc201580690)

[S1 Flowchart highlighting different subgroups of analysis. 3](#_Toc201580691)

[S2 Area under the curve of the skin perfusion expressed as CVC during cooling and rewarming for the three groups 4](#_Toc201580692)

[S3 Venn diagram of the differential gene expression between the three groups 5](#_Toc201580693)

[S4 Representation of the nitric oxide stimulates guanylate cyclase pathway (HAS-392154) 6](#_Toc201580694)

[S5 Representation of the extracellular matrix organisation pathway 7](#_Toc201580695)

[Supplemental tables 9](#_Toc201580696)

[Table S1: Primers sequences used for RT-qPCR 9](#_Toc201580697)

[Table S2: Mean cutaneous vascular conductance (CVC +/- sd) 10](#_Toc201580698)

[Table S3: Mean AUC +sd 13](#_Toc201580699)

[Table S4: Shared pathways between Healthy subjects and patients with PRP versus patients with RP-SSC 14](#_Toc201580700)

[Supplemental data 15](#_Toc201580701)

## Supplemental figures

S1 Flowchart highlighting different subgroups of analysis.


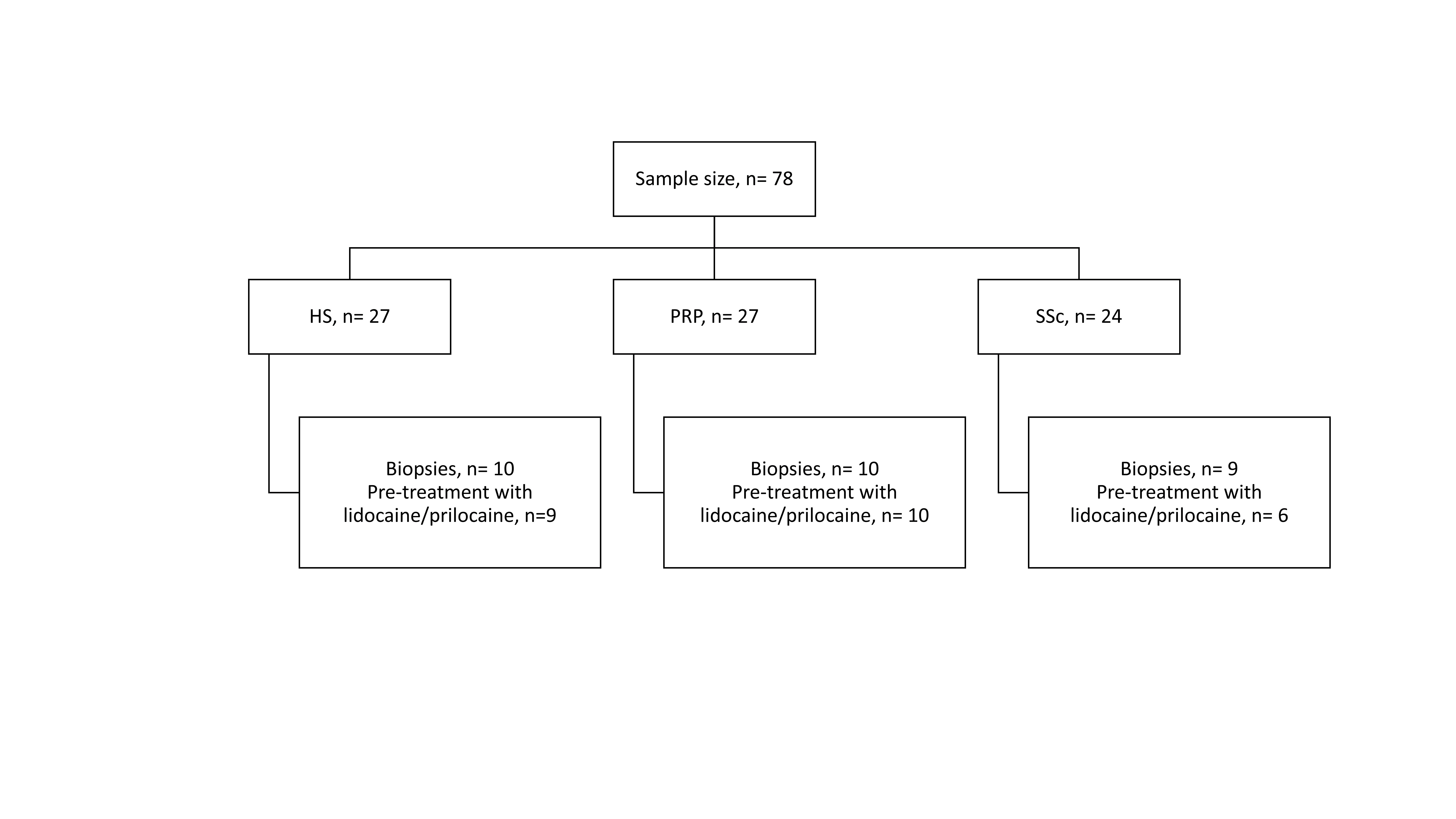


Skin blood flux was analysed on 3 fingers for subjects who had only undergone cooling test, on 2 fingers for subjects with one finger pre-treated with lidocaine/prilocaine and one finger only for subjects who had undergone biopsies. Fingers pretreated with lidocaine/prilocaine were not analysed.

HS= Healthy subjects, PRP = Primary Raynaud Phenomenon, SSc = Systemic Sclerosis

### S2 Area under the curve of the skin perfusion expressed as CVC during cooling and rewarming for the three groups

CVC expressed as AU/mmHg


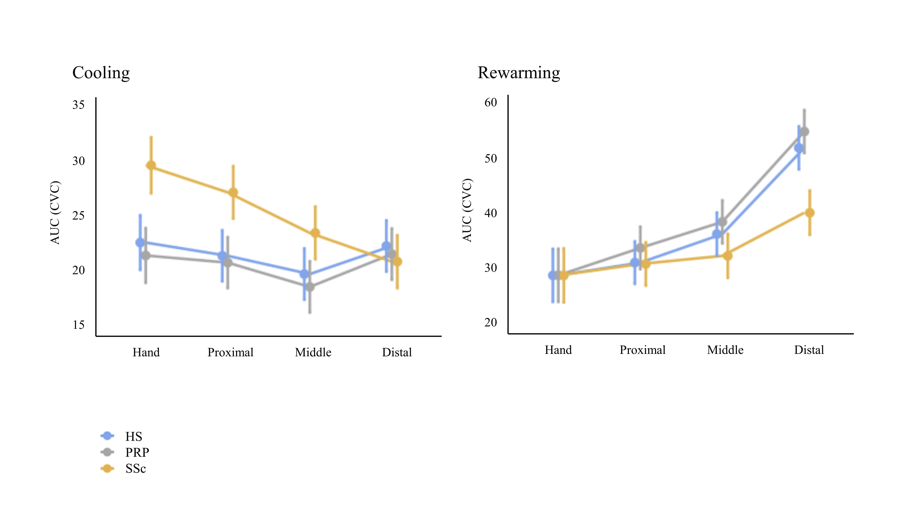


### S3 Venn diagram of the differential gene expression between the three groups


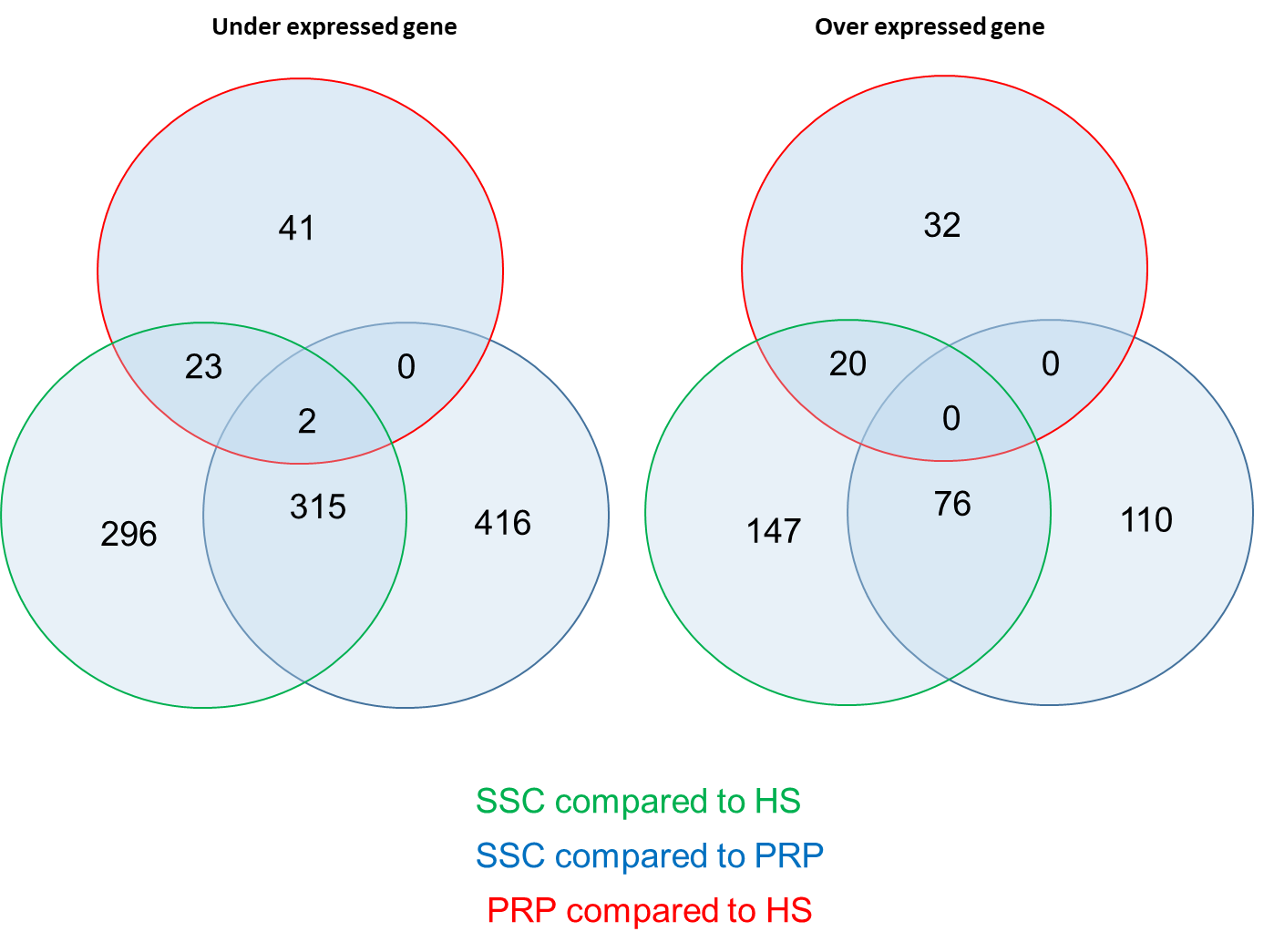


HS= Healthy subjects, PRP = Primary Raynaud Phenomenon, SSc = Systemic Sclerosis


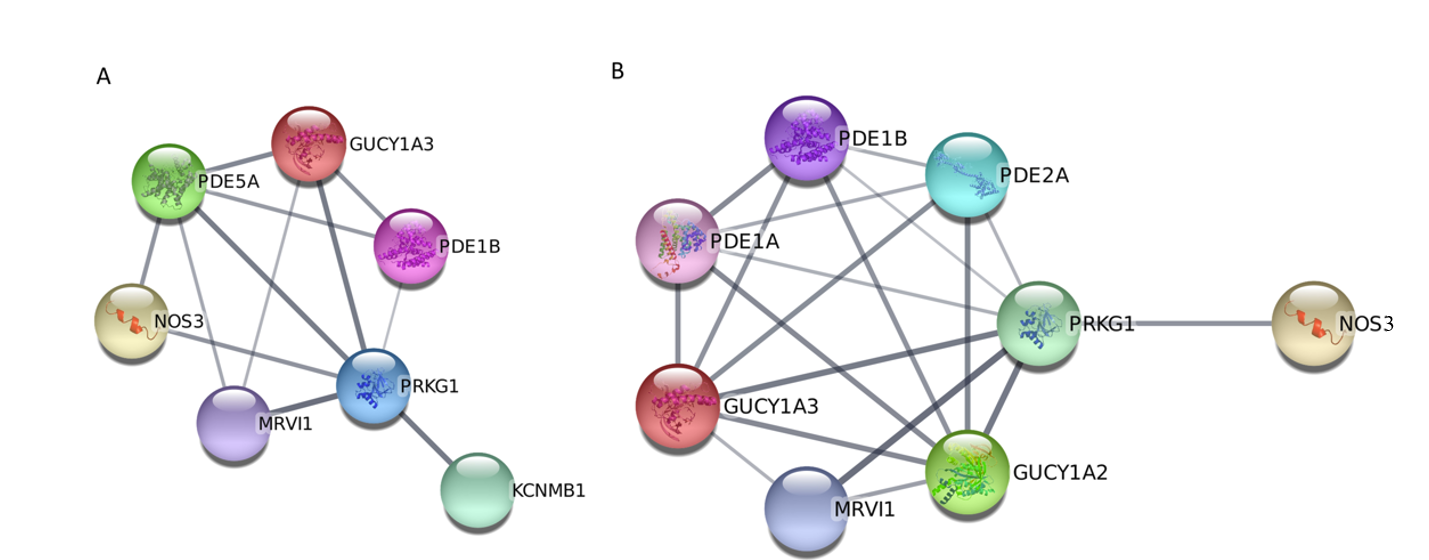
S4 Representation of the nitric oxide stimulates guanylate cyclase pathway (HAS-392154)

A: SSc-RP versus HS B: SSc-RP versus PR

S5 Representation of the extracellular matrix organisation pathway


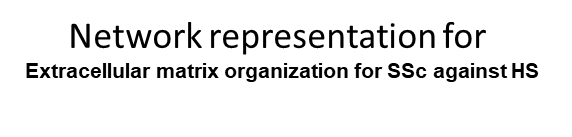

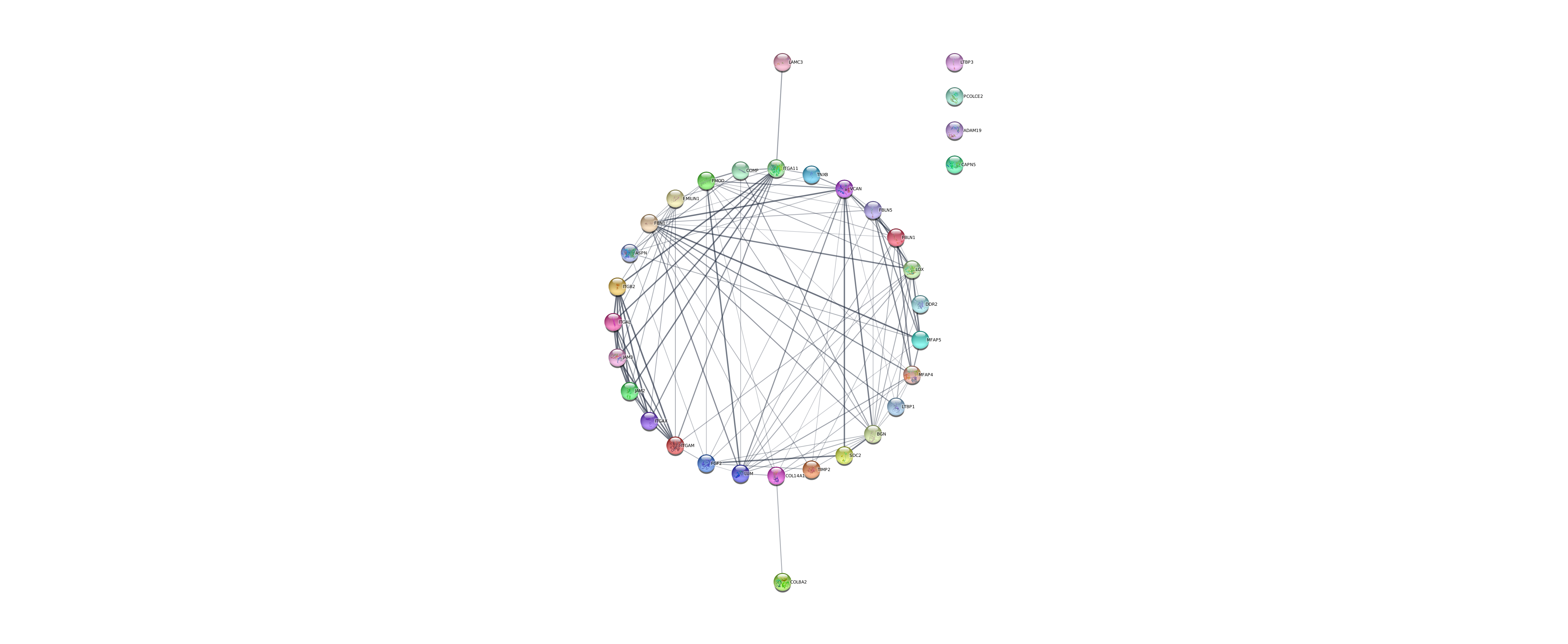


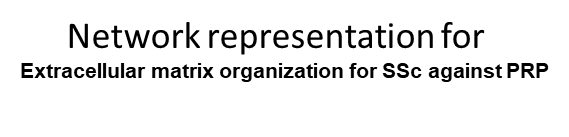


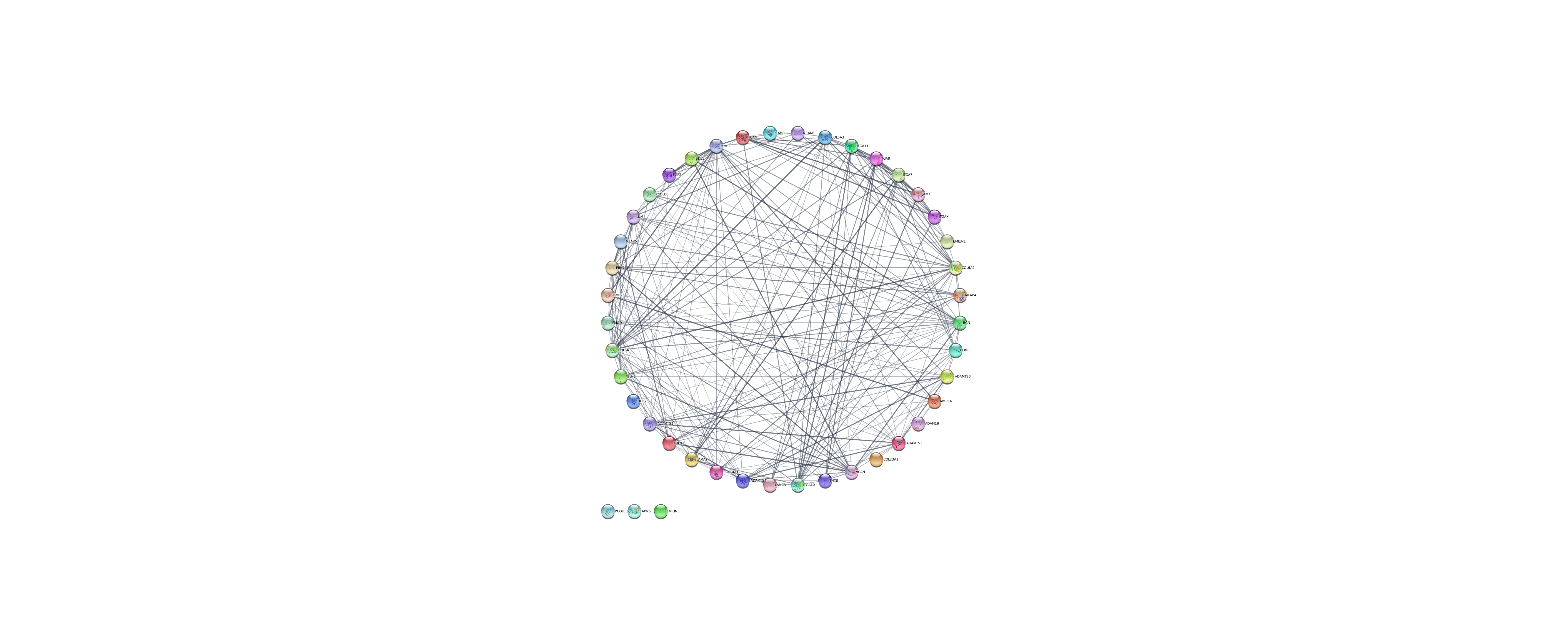


## Supplemental tables

### Table S1A: Primers sequences used for RT-qPCR (TRPs)


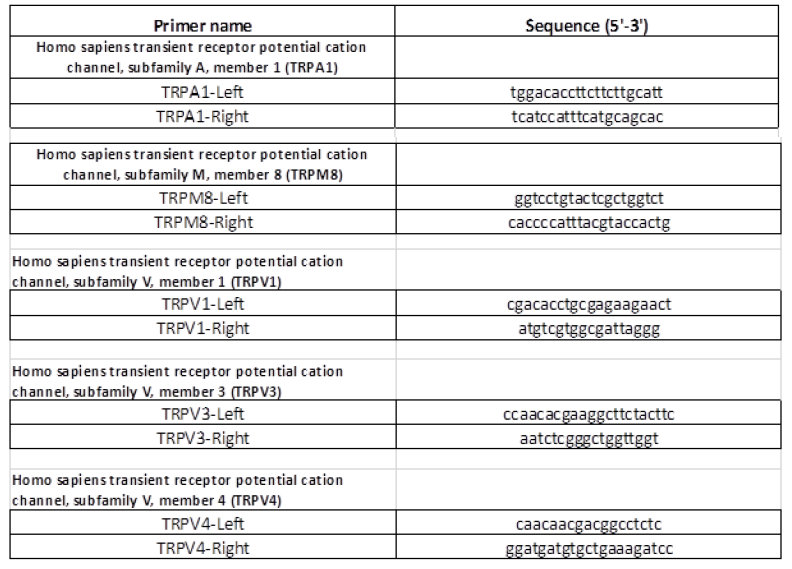


### Table S1B: Primers sequences used for RT-qPCR (Housekeeping gene)

| Homo sapiens Ribosomal Protein L27 | Sequence (5’-3’) |
| --- | --- |
| RPL27-Left | tggacaaaactgtcgtcaataagg |
| RPL27-Right | agaaccacttgttcttgcctgtc |

### Table S2: Mean cutaneous vascular conductance (CVC +/- sd)

|  |  |  |  |  |  |  |  | |  |  |  |  |  |
| --- | --- | --- | --- | --- | --- | --- | --- | --- | --- | --- | --- | --- | --- |
|  |  | **Healthy** |  | **Primary RP** |  | **Secondary RP** | **P-Value** | | | | | |  |
|  | n | Mean ± sd | n | Mean ± sd | n | Mean ± sd | Site | Group | | Site-group interaction | Time-site interaction | Time-group interaction | Time-site-group interaction |
| Overall |  |  |  |  |  |  | <0.001 | 0.963 | | <0.001 | <0.001 | <0.001 | 0.008 |
| Hand | 259 | 0.57 ± 0.24 | 264 | 0.57 ± 0.27 | 230 | 0.70 ± 0.42 |  | 0.163 | |  |  |  |  |
| Proximal | 633 | 0.58 ± 0.24 | 638 | 0.61 ± 0.28 | 582 | 0.71 ± 0.45 |  | 0.396 | |  |  |  |  |
| Medial | 534 | 0.62 ± 0.25 | 528 | 0.65 ± 0.33 | 516 | 0.73 ± 0.52 |  | 0.765 | |  |  |  |  |
| Distal | 534 | 0.87 ± 0.44 | 528 | 0.87 ± 0.53 | 516 | 0.81 ± 0.57 |  | 0.017*# | |  |  |  |  |
|  |  |  |  |  |  |  |  |  | |  |  |  |  |
| CVC Baseline |  |  |  |  |  |  |  |  | |  |  |  |  |
| Hand | 27 | 0.65 ± 0.21 | 27 | 0.68 ± 0.31 | 24 | 0.78 ± 0.36 |  | 0.483 | |  |  |  |  |
| Proximal | 61 | 0.65 ± 0.17 | 61 | 0.68 ± 0.27 | 56 | 0.89 ± 0.53 |  | 0.060 | |  |  |  |  |
| Medial | 52 | 0.71 ± 0.2 | 51 | 0.76 ± 0.36 | 50 | 1.02 ± 0.67 |  | 0.073 | |  |  |  |  |
| Distal | 52 | 1.06 ± 0.41 | 51 | 0.97 ± 0.57 | 50 | 1.17 ± 0.64 |  | 0.191 | |  |  |  |  |
| CVC Cooling test beginning |  |  |  |  |  |  |  |  | |  |  |  |  |
| Hand | 27 | 0.77 ± 0.29 | 27 | 0.82 ± 0.39 | 24 | 1.07 ± 0.57 |  | 0.185 | |  |  |  |  |
| Proximal | 61 | 0.8 ± 0.3 | 61 | 0.85 ± 0.38 | 56 | 1.1 ± 0.59 |  | 0.080 | |  |  |  |  |
| Medial | 52 | 0.78 ± 0.29 | 51 | 0.76 ± 0.34 | 50 | 1.15 ± 0.67 |  | 0.035# | |  |  |  |  |
| Distal | 52 | 0.95 ± 0.39 | 51 | 0.83 ± 0.41 | 50 | 1.06 ± 0.59 |  | 0.154 | |  |  |  |  |
| CVC Cooling test 10minutes |  |  |  |  |  |  |  |  | |  |  |  |  |
| Hand | 27 | 0.69 ± 0.27 | 27 | 0.69 ± 0.32 | 23 | 0.97 ± 0.53 |  | 0.086 | |  |  |  |  |
| Proximal | 61 | 0.7 ± 0.29 | 61 | 0.69 ± 0.34 | 55 | 0.95 ± 0.53 |  | 0.035# | |  |  |  |  |
| Medial | 52 | 0.68 ± 0.26 | 51 | 0.62 ± 0.3 | 49 | 0.9 ± 0.56 |  | 0.086 | |  |  |  |  |
| Distal | 52 | 0.8 ± 0.33 | 51 | 0.7 ± 0.34 | 49 | 0.79 ± 0.38 |  | 0.345 | |  |  |  |  |
| CVC Cooling test 20minutes |  |  |  |  |  |  |  |  | |  |  |  |  |
| Hand | 27 | 0.62 ± 0.26 | 27 | 0.62 ± 0.29 | 23 | 0.88 ± 0.45 |  | 0.068 | |  |  |  |  |
| Proximal | 61 | 0.64 ± 0.28 | 61 | 0.61 ± 0.27 | 55 | 0.87 ± 0.46 |  | 0.011# | |  |  |  |  |
| Medial | 52 | 0.61 ± 0.26 | 51 | 0.55 ± 0.26 | 49 | 0.76 ± 0.39 |  | 0.070 | |  |  |  |  |
| Distal | 52 | 0.72 ± 0.25 | 51 | 0.64 ± 0.32 | 49 | 0.75 ± 0.4 |  | 0.331 | |  |  |  |  |
| CVC Cooling test 30mintes |  |  |  |  |  |  |  |  | |  |  |  |  |
| Hand | 27 | 0.57 ± 0.24 | 27 | 0.57 ± 0.27 | 23 | 0.7 ± 0.38 |  | 0.453 | |  |  |  |  |
| Proximal | 61 | 0.6 ± 0.25 | 61 | 0.59 ± 0.31 | 55 | 0.7 ± 0.37 |  | 0.431 | |  |  |  |  |
| Medial | 52 | 0.59 ± 0.23 | 51 | 0.59 ± 0.42 | 49 | 0.61 ± 0.35 |  | 0.539 | |  |  |  |  |
| Distal | 52 | 0.72 ± 0.26 | 51 | 0.75 ± 0.6 | 49 | 0.61 ± 0.37 |  | 0.033* | |  |  |  |  |
| CVC Exit of the box |  |  |  |  |  |  |  |  | |  |  |  |  |
| Hand | 17 | 0.39 ± 0.09 | 17 | 0.43 ± 0.11 | 16 | 0.41 ± 0.12 |  | 0.753 | |  |  |  |  |
| Proximal | 51 | 0.43 ± 0.1 | 51 | 0.53 ± 0.2 | 48 | 0.44 ± 0.17 |  | 0.239 | |  |  |  |  |
| Medial | 42 | 0.5 ± 0.15 | 41 | 0.65 ± 0.33 | 42 | 0.41 ± 0.23 |  | <0.001*# | |  |  |  |  |
| Distal | 42 | 0.87 ± 0.37 | 41 | 1 ± 0.63 | 42 | 0.48 ± 0.4 |  | < 0.001*# | |  |  |  |  |
| CVC 10 minutes post cooling |  |  |  |  |  |  |  |  | |  |  |  |  |
| Hand | 17 | 0.37 ± 0.07 | 18 | 0.42 ± 0.12 | 16 | 0.41 ± 0.13 |  | 0.620 | |  |  |  |  |
| Proximal | 51 | 0.41 ± 0.1 | 52 | 0.52 ± 0.2 | 48 | 0.41 ± 0.15 |  | 0.060 | |  |  |  |  |
| Medial | 42 | 0.48 ± 0.18 | 42 | 0.65 ± 0.36 | 42 | 0.38 ± 0.21 |  | <0 .001*# | |  |  |  |  |
| Distal | 42 | 0.76 ± 0.44 | 42 | 1.07 ± 0.72 | 42 | 0.46 ± 0.38 |  | < 0.001*#$ | |  |  |  |  |
| CVC 20 minutes post cooling |  |  |  |  |  |  |  |  | |  |  |  |  |
| Hand | 22 | 0.41 ± 0.09 | 23 | 0.43 ± 0.1 | 23 | 0.52 ± 0.26 |  | 0.469 | |  |  |  |  |
| Proximal | 56 | 0.47 ± 0.12 | 57 | 0.52 ± 0.16 | 55 | 0.48 ± 0.21 |  | 0.518 | |  |  |  |  |
| Medial | 47 | 0.55 ± 0.2 | 47 | 0.62 ± 0.21 | 49 | 0.53 ± 0.33 |  | 0.021# | |  |  |  |  |
| Distal | 47 | 0.87 ± 0.53 | 47 | 0.95 ± 0.49 | 49 | 0.71 ± 0.57 |  | <0.001*# | |  |  |  |  |
| CVC 30 minutes post cooling |  |  |  |  |  |  |  |  | |  |  |  |  |
| Hand | 26 | 0.48 ± 0.14 | 27 | 0.48 ± 0.16 | 24 | 0.54 ± 0.21 |  | 0.792 | |  |  |  |  |
| Proximal | 60 | 0.52 ± 0.17 | 61 | 0.58 ± 0.25 | 56 | 0.57 ± 0.25 |  | 0.653 | |  |  |  |  |
| Medial | 51 | 0.6 ± 0.24 | 51 | 0.71 ± 0.42 | 50 | 0.64 ± 0.41 |  | 0.120 | |  |  |  |  |
| Distal | 51 | 0.94 ± 0.57 | 51 | 1.01 ± 0.6 | 50 | 0.91 ± 0.64 |  | 0.066 | |  |  |  |  |
| CVC 40 minutes post cooling |  |  |  |  |  |  |  |  | |  |  |  |  |
| Hand | 25 | 0.53 ± 0.18 | 27 | 0.51 ± 0.19 | 18 | 0.56 ± 0.24 |  | 0.932 | |  |  |  |  |
| Proximal | 59 | 0.57 ± 0.19 | 61 | 0.57 ± 0.22 | 50 | 0.62 ± 0.32 |  | 0.806 | |  |  |  |  |
| Medial | 50 | 0.65 ± 0.26 | 51 | 0.62 ± 0.27 | 44 | 0.7 ± 0.44 |  | 0.833 | |  |  |  |  |
| Distal | 50 | 0.99 ± 0.55 | 51 | 0.88 ± 0.45 | 44 | 0.91 ± 0.68 |  | 0.459 | |  |  |  |  |
| CVC 60 minutes post cooling |  |  |  |  |  |  |  |  | |  |  |  |  |
| Hand | 17 | 0.57 ± 0.25 | 17 | 0.48 ± 0.14 | 16 | 0.63 ± 0.3 |  | 0.228 | |  |  |  |  |
| Proximal | 51 | 0.55 ± 0.18 | 51 | 0.56 ± 0.18 | 48 | 0.73 ± 0.37 |  | 0.025* | |  |  |  |  |
| Medial | 42 | 0.61 ± 0.23 | 41 | 0.59 ± 0.23 | 42 | 0.85 ± 0.57 |  | 0.045& | |  |  |  |  |
| Distal | 42 | 0.87 ± 0.47 | 41 | 0.76 ± 0.39 | 42 | 1 ± 0.65 |  | 0.189 | |  |  |  |  |
|  |  |  |  |  |  |  |  |  | |  |  |  |  |

CVC in AU/mmHg

* Significant post-hoc p value adjusted healthy versus secondary RP

# Significant post-hoc p value adjusted primary versus secondary RP

$ Significant post-hoc p value adjusted healthy versus primary RP

& Non-significant post-hoc p value adjusted primary versus secondary RP (p-value adjusted = 0.054)

### Table S3: Mean AUC +sd

|  |  |  |  |  |  |  |  |  |  |
| --- | --- | --- | --- | --- | --- | --- | --- | --- | --- |
|  |  | **Healthy** |  | **Primary RP** |  | **Secondary RP** | **P-Value** | | |
|  | n | Mean ± sd | n | Mean ± sd | n | Mean ± sd | Site | Group | Site-group interaction |
| **AUC CVC Cooling** |  |  |  |  |  |  | <0.001 | 0.752 | <0.001 |
| Hand | 17 | 22.1 ± 7.94 | 17 | 20.8 ± 10 | 16 | 29.4 ± 14.8 |  | 0.244 |  |
| Proximal | 51 | 20.9 ± 8.36 | 51 | 20.3 ± 9.49 | 48 | 27.2 ± 13.9 |  | 0.463 |  |
| Medial | 42 | 20.3 ± 7.65 | 41 | 18.7 ± 9.39 | 42 | 25.6 ± 12.8 |  | 0.743 |  |
| Distal | 42 | 23.6 ± 7.37 | 41 | 22.1 ± 10.6 | 42 | 23.3 ± 9.8 |  | 0.603 |  |
| **AUC CVC Rewarming** |  |  |  |  |  |  | <0.001 | 0.294 | <0.001 |
| Hand | 17 | 26.8 ± 5.83 | 17 | 26.4 ± 6.38 | 16 | 28.2 ± 9.55 |  | 0.975 |  |
| Proximal | 51 | 29.2 ± 7.37 | 51 | 31.8 ± 8.82 | 48 | 31.4 ± 12.5 |  | 0.623 |  |
| Medial | 42 | 33.6 ± 11.5 | 41 | 36.8 ± 12.9 | 42 | 34.0 ± 18.0 |  | 0.199 |  |
| Distal | 42 | 52.4 ± 27.2 | 41 | 55.2 ± 23.3 | 42 | 43.0 ± 26.7 |  | 0,010# |  |

* Significant post-hoc p value adjusted healthy versus secondary RP

# Significant post-hoc p value adjusted primary versus secondary RP

### Table S4: Shared pathways between Healthy subjects and patients with PRP versus patients with RP-SSC

| Under expressed | | | |
| --- | --- | --- | --- |
| description | term name | FDR value  (SSc vs HS) | FDR value  (SSc-RP vs PRP ) |
| Extracellular matrix organization | HSA-1474244 | 1.32E-07  * | 4.69E-12  $ |
| Nitric oxide stimulates guanylate cyclase | HSA-392154 | 5.40E-03 | 1.10E-03 |

HSA: Reactomes pathway ; SSc: patients with systemic sclerosis; HS:healthy subjects, PRP: patients with primary Raynaud Phenomenon

* *ADAM19, ASPN, BGN, CAPN5, COL14A1, COL8A2, COMP, DDR2, EMILIN1, FBLN1, FBLN5, FBN1, FGF2, FMOD, ITGA11, ITGAL, ITGAM, ITGAX, ITGB2, JAM2, JAM3, LAMC3, LOX, LTBP1, LTBP3, LUM, MFAP4, MFAP5, PCOLCE2, SDC2, TIMP2, TNXB, VCAN*

*$ ADAM19, ADAMTS2, ADAMTS3, ADAMTS4, ADAMTS5, BGN, CAPN5, COL14A1, COL23A1, COL6A1, COL6A2, COL6A3, COMP, DDR2, EMILIN1, EMILIN3, FBLN1, FBLN2, FBN1, FGF2, FMOD, ICAM3, ITGA10, ITGA11, ITGA7, ITGA8, ITGAM, ITGAX, JAM3, LAMA2, LAMC3, LUM, MFAP4, MFAP5, MMP16, MMP2, NCAM1, PCOLCE, PCOLCE2, SDC2, TIMP2, TNXB, VCAN*

## Supplemental data

Supplemental data S1 and S2 are table on separate files.
